# Effects of air-conditioning systems in the public areas of hospitals: A scoping review

**DOI:** 10.1017/S0950268821001990

**Published:** 2021-08-27

**Authors:** Han Ting Wu, Qiu Shuang Li, Rong Chen Dai, Shan Liu, Li Wu, Wei Mao, Cong Hua Ji

**Affiliations:** 1 College of Public Health, Zhejiang Chinese Medical University, Hangzhou, China; 2 The First Affiliated Hospital of Zhejiang Chinese Medical University, Hangzhou, China

**Keywords:** Air-conditioning systems, COVID-19, hospital, pathogenic microorganisms, public area, AC, air-conditioning systems, HVAC, heating, ventilation and air-conditioning, HEPA, high-efficiency particulate air, ICU, intensive care unit, CFU, colony forming unit, CO, carbon monoxide, IPM, invasive pulmonary mycoses, PM, particulate matter, LAF, laminar airflow, IA, invasive aspergillosis, TB, tubercle bacillus, ACH, air changes per hour

## Abstract

Almost all hospitals are equipped with air-conditioning systems to provide a comfortable environment for patients and staff. However, the accumulation of dust and moisture within these systems increases the risk of transmission of microbes and have on occasion been associated with outbreaks of infection. Nevertheless, the impact of air-conditioning on the transmission of microorganisms leading to infection remains largely uncertain. We conducted a scoping review to screen systematically the evidence for such an association in the face of the coronavirus disease 2019 epidemic. PubMed, Embase and Web of Science databases were explored for relevant studies addressing microbial contamination of the air, their transmission and association with infectious diseases. The review process yielded 21 publications, 17 of which were cross-sectional studies, three were cohort studies and one case−control study. Our analysis showed that, compared with naturally ventilated areas, microbial loads were significantly lower in air-conditioned areas, but the incidence of infections increased if not properly managed. The use of high-efficiency particulate air (HEPA) filtration not only decreased transmission of airborne bioaerosols and various microorganisms, but also reduced the risk of infections. By contrast, contaminated air-conditioning systems in hospital rooms were associated with a higher risk of patient infection. Cleaning and maintenance of such systems to recommended standards should be performed regularly and where appropriate, the installation of HEPA filters can effectively mitigate microbial contamination in the public areas of hospitals.

## Introduction

The outbreak of the novel severe acute respiratory syndrome coronavirus 2 disease (SARS-CoV-2), the cause of coronavirus disease 2019 (COVID-19), has currently spread to almost all parts of the world. Available evidence indicates that the agent is transmitted via respiratory droplets and contact routes between humans [1]. Measures that hinder the spread of the virus include environmental control of indoor air flow [2]. However, relatively little attention has been paid to air-conditioning systems, which are one of the most common factors affecting indoor air flow. Some reports have implicated such systems in the transmission of SARS-CoV-2 [3,4], and norovirus [5].

Air-conditioning systems play an important role in maintaining indoor air temperature and humidity in public buildings and hospitals. In the latter, particularly, intensive care units (ICUs) and operating rooms, the systems are fitted with high-efficiency particulate air (HEPA) filtration and laminar flow design to reduce the risk of air-borne infections. Installation of air-conditioning systems can help prevent hyperthermia in critically ill infected patients in a heat wave, and may reduce the cost of blood cultures requested since the number of cultures taken increases in such patients if a high ambient temperature is sustained [6]. The systems have also proven effective in reducing mortality in heat-related illness in domestic homes [7], and hospitals [8].

However, air-conditioning systems represent a potential source of microbial contamination in hospitals, as accumulated dust and moisture increase the risk of contamination and associated infections. Indeed, several fungal genera have been demonstrated in air-conditioned ICU [9], and mould colonisation has been observed in HEPA filters, and in air-conditioning systems [10], as has the presence of SARS-CoV-2 on swab samples taken from surfaces of filters [11]. Likewise, contamination of air-conditioning systems has been implicated in some hospital-acquired infections [12–14].

The risk of proliferation of microbes from air-conditioning systems and their transmission to high-risk patients, in hospitals is greatly reduced if strict management and control practices are followed [15]. However, despite the several regulations covering the installation of these systems in hospitals, such as the HVAC Design Manual for Hospitals and Clinics published by the American Society of Heating, Refrigeration and Air-Conditioning Engineers, adherence to these standards is variable in routine practice. Indeed, epidemiological surveillance in a hospital in Paris found that only 32% of the patients diagnosed with invasive nosocomial aspergillosis were housed in rooms where an HEPA air filter system had been installed [16].

Studies on heating, ventilation and air-conditioning (HVAC) systems in hospitals have largely been conducted in restricted areas such as operating rooms and ICUs, and have focused on the impact of different airflow patterns, number of personnel, ventilation rates and other extrinsic factors [17–19]. However, in the public areas of hospitals (wards, clinics etc.) airflow may be suboptimal and result in a higher risk of microbial contamination. This study therefore focused on these areas in which high-efficiency filters are rarely installed, and which have often been overlooked in other investigations.

Our aim was to clarify the presence and nature of potential risks associated with the use of air-conditioning systems, through the systematic assembly and analysis of published evidence on the effect of air-conditioning systems on the transmission of pathogens and related infectious diseases. Further, we explored effective measures for the protection of patients, staff and visitors from the potential risks of exposure to microorganisms related to air-conditioning systems, and application of measures with potential to combat such transmission in the COVID-19 pandemic.

## Methods

The guidelines of the Preferred Reporting Items for Systematic Reviews and Meta-Analyses Extension for Scoping Reviews (PRISMA-ScR) [20] were followed in this research study. The key stages of this framework [21] were: identifying the research question, identifying relevant studies, study selection, charting of data and collating, summarising and reporting the results.

### Research questions

The study addressed the following questions:

1. Is there any association between air-conditioning systems and the presence of pathogenic microorganisms in public areas of hospitals?
2. Do air-conditioning systems increase the risk of infection in such hospital areas?

### Relevant studies

The complete PubMed, Embase and Web of Science databases were explored for relevant studies in December 2020. The search strategy is outlined in ‘Supplementary Material’. Papers published since the databases were established were included in the search, and relevant cited references.

### Inclusion and exclusion criteria

All studies met the following criteria: published in English; intervention included different kinds of air-conditioning systems, such as unfiltered air or air-conditioning systems with HEPA filters; comparators were other areas without air-conditioning systems; assessment of the presence and measurement of pathogenic microorganisms in indoor air, ratios of viable microorganisms detected, incidence of infectious diseases, among others. The year of publication was not restricted in the literature search.

The exclusion criteria included the following: air-conditioning systems located only in operating rooms or other restricted areas; additional interventions (e.g. ultraviolet germicidal irradiation) combined with air-conditioning but focused on outcomes irrelevant to air-conditioning; and studies lacking specific data or comparators.

### Study selection

All articles identified in the databases were exported into Endnote (Version 9.3), and duplicates were removed on initial screening. Study titles and abstracts and web searches of citations of relevant studies were screened by two independent researchers (Han-Ting Wu and Rong-Chen Dai) to assess their potential relevance for full review. The same researchers also independently reviewed the full texts of candidate articles against the inclusion and exclusion criteria. Any discrepancies were resolved through discussion with a third reviewer. The reasons for exclusion during the screening of the full texts were recorded.

### Charting the data

Data were extracted independently by two review authors and discrepancies were identified and resolved as above. They comprised: names of the authors, year, type of study, outcome of interest, bacterial or fungal pathogens, hospital locations tested, air-conditioning systems used and relevant results and study conclusion.

### Collating, summarising and reporting results

Due to the heterogeneity of studies and difficulty of quantifying the data, we tabulated key information i.e. the kinds of air-conditioning systems, tested areas and study designs and described relevant parameters in detail. Quantitative and qualitative findings were summarised within each grouping of air-conditioning systems and related quantitative data such as the concentrations of microorganisms found in samples of indoor air were recorded. Associations are presented using the summary measures reported in individual studies with *P*-values where available

## Results

### Selection of studies

**Fig. 1.**
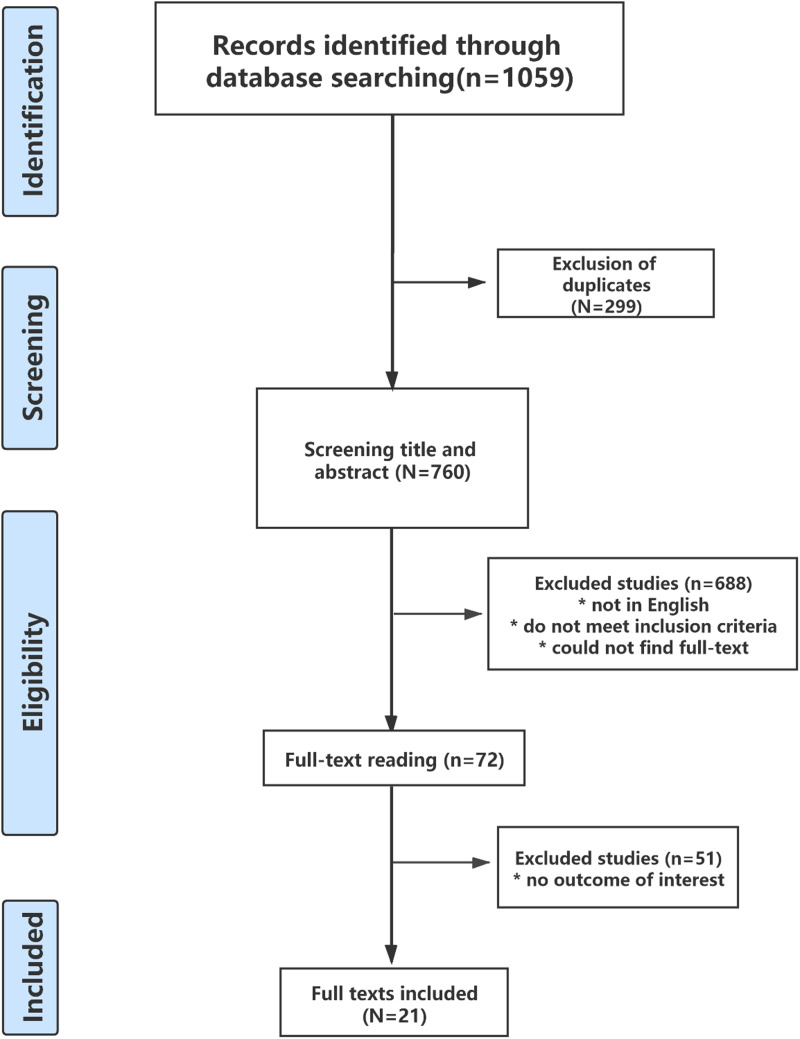
PRISMA diagram of the screening and selection process.

Figure 1 presents the PRISMA diagram for the screening and selection of articles. A total of 1059 studies were retrieved, of which 299 duplicates and 688 irrelevant studies were excluded often because either they were not reported in English, did not meet the inclusion criteria, or their full texts were not available. As a consequence, 72 studies were assessed for eligibility; 51 were excluded as ancillary disinfection equipment was used along with air-conditioning systems, or only samples taken from air conditioners were tested, or evidence of the effect of air-conditioning systems or comparators was lacking. This process left 21 articles for analysis [22–42]. Seventeen were cross-sectional studies, three were cohort studies and one was a case−control study. All, but one, were published after 2000 and the other in 1975. Most of the articles were published in internationally recognised and specialised journals; an overview of the articles and their outcomes is presented in Table 1.

**Table 1.** General overview of the studies

Authors	Study	Microbes/Infection	Locations	Air-conditioning systems ((I): intervention; (C): control)	Key findings	Conclusions
Perdelli, *et al*., [22] 2006	Cross-sectional	Bacteria and fungi	Wards	**(I)**:Common AC **(C1)**:Natural ventilation **(C2)**:With HEPA filters	The ward with no air-conditioning system (A) had the worst results on all three types of sampling carried out; the total bacterial load and the sedimented mycotic load were almost twice as high as the values recorded in the ward with the system without HEPA filters (B). The percentage of samples positive for air-borne *Aspergillus* was also twice as high in A as in B.	Air-conditioning systems markedly reduce the concentration of aspergilli in the environment.
Sornboot, *et al*. [23] 2019	Cross-sectional	Bacteria and fungi	Emergency department TB ward/clinic Bronchoscopy unit	**(I)**: Common AC (Split-type) **(C)**: hybrid ventilation	Air-conditioning systems used in the areas were mostly split-type (44%) and central-type air conditioners (48%). Multiple linear regression analysis showed that the concentration of indoor air-borne bioaerosols was positively correlated with ventilation system (e.g. central-type air conditioner) (*P* < 0.05).	Improved air change rate and avoiding use of central-type air-conditioning systems may reduce bioaerosol concentrations.
Çakir, *et al*.. [24] 2013	Cross-sectional	Bacteria and fungi	Wards, corridors, Operating theatres and postoperative units	**(I)**: Common AC **(C)**: With HEPA filters	While the number of microorganisms collected in hospital 2 before the disinfection process was higher than those after the disinfection process, this was reversed in hospital 1. In the latter, the air-conditioning system and the HEPA filters which were switched on before the disinfection process, were turned off during the weekend, and thus the number of airborne live microorganisms increased fivefold after the disinfection process.	Microbial loads in the hospital air were effectively controlled due to use of HEPA filters in air-conditioning systems.

Authors	Study	Microbes/Infection	Locations	Air-conditioning systems (I): intervention; (C): control	Key findings	Conclusions
Crimi *et al*. [25] 2009	Cross-sectional	Bacteria and fungi	Wards	**(I)**: Common AC **(C)**: With HEPA filters	Unit A, without HEPA filters showed a gradual increase in the bacterial load 20 and 60 days after cleaning of the ventilation system. No fungi were present in basal conditions, at 20 or 60 days after decontamination. Unit B, equipped with HEPA filters placed at the inlet vents, showed extremely low bacterial loads either in basal conditions or upon inspection 60 days after cleaning.	Units with ventilation equipment lacking HEPA filters maintain emission of air uncontaminated by fungi for 2 months. HEPA is not necessary if ventilation ducts are frequently cleaned.
Perdelli *et al*. [26] 2006	Cross-sectional	Fungi	Operating theatre Other: wards, laboratories, outpatient departments, kitchens, etc. Outdoor	**(I)** Common AC (Filtering efficiency of 80%−85%) **(C1)**:Natural ventilation **(C2)**:With HEPA filters	Statistically significant differences in fungal concentrations (rank comparison) in various environments (*χ*^2^ = 58.226, *P* < 0.001. Genera implicated in infections were : *Aspergillus*, *Penicillium*, *Cladosporium* and *Rhizopus*.	Fungal concentrations recorded are comparable to other studies in hospital environments and considerably lower than in other indoor environments without air conditioning. Air-conditioning systems are effective in reducing fungal contamination.

Authors	Study	Microbes/Infection	Locations	Air-conditioning systems ((I): intervention; (C): control)	Key findings	Conclusions
Lidwell and Noble [27] 1975	Cross-sectional	Fungi	Hospital Outdoor	**(I)**: Common AC **(C)**: Natural ventilation	The numbers of fungi growing at 21°C in the air-conditioned hospital were less than 10% of those recorded from the control hospital.	Most airborne fungi in naturally ventilated hospital wards are probably introduced with the ventilating air.
Crimi *et al*. [28] 2006	Cross-sectional	Bacteria and fungi	Wards	**(I)**: With HEPA filters (central/ peripheral) **(C)**: Natural ventilation	60% of samples taken at emission outlets in six departments yielded a positive result. *Aspergillus* levels from naturally ventilated rooms were markedly higher than in departments with mechanical ventilation. The corresponding recovery rates for bacteria were 83% and (38.6%) (*χ*^2^ = 9.778, *P* = 0.002). Similarly, *Aspergillus* rates in departments with central filters were higher than those with peripheral filters (4.4% vs. 1.2%). In contrast, bacterial loads introduced in departments with central filters were significantly higher (58.2 CFU/m^3^) compared with peripheral filters (21.4 CFU/m^3^)(*t*-Tes*t* = 5.033, *P* < 0.001).	The presence of artificial ventilation systems can lower bacterial and fungal concentrations compared with natural ventilation.

Authors	Study	Microbes/Infection	Locations	Air-conditioning systems (I): intervention; (C): control)	Key findings	Conclusions
Cho *et al*. [29] 2018	Cross-sectional	Fungi	Wards Cleanroom (patients lounge’) Outdoor	**(I)**:Common AC **(C1)**:Natural ventilation **(C2)**:With HEPA filters	Concordant with previous results, mean fungal counts inside the hospital and clean area was 26.5 CFU/m^3^ and < 5 CFU/m^3^, respectively.	AC and HEPA filtering systems should be used to reduce fungal counts in hospitals to prevent infections.
Falvey and Streifel .[30] 2007	Cross-sectional	Fungi	Bone Marrow Transplant unit Patient care areas (PCAs) Indoor reference	**(I)**: Common AC (filtering efficiency of 65%/ 90%–95%) **(C)**: With HEPA filters	Outdoor reference samples yielded >40 times more CFU/m^3^ than PCAs with HEPA-filtered fans, and > 25 times more CFU/m^3^ than other patient care units. Samples from PCAs lacking HEPA-filtered air gave almost indistinguishable counts of *Aspergillus* spp. from other reference samples but all PCAs had significantly less samples positive for *Aspergillus fumigatus* compared with outdoor samples.	Emphasis should be placed on maintaining high-efficiency filtration of outside air and on ensuring that other environmental control methods are used to prevent dissemination of environmental opportunistic fungal spores.
Kruger *et al*. [31] 2003	Cross-sectional	Fungi	Patient rooms Outside Corridor	**(I)**: Common AC **(C1)**: Unknown **(C2)**: With HEPA filters	Mean fungal counts in corridor air (I) were significantly higher than in patient rooms (C2) prior to and during building construction (*P* < 0.05).	HEPA filtration with locked rooms and positive pressure between corridor and patient rooms is sufficient to protect patients against fungal spores liberated by construction in neighbourhood.

Authors	Study	Microbes/Infection	Locations	Air-conditioning systems used ((I): intervention; (C): control)	Key findings	Conclusions
Bellanger *et al*. [32] 2017	Cross-sectional	Fungi Exposure risk	Corridors Wards Control room	**(I)**: Common AC **(C1)**: With low air pressure (B2) **(C2)**: With HEPA filters (B3)	Lower quantities of opportunistic fungi were detected in rooms equipped with laminar airflow (40 volumes per hour, rooms with positive 25-Pa pressure and HEPA filters) than in control rooms. Building 2 (C1) was approximately 2.9 times more contaminated by moulds than building 3 (C2) (*P* = 0.004), whereas B1 was approximately 1.8 times more contaminated than building 3(I) (*P* = 0.32).	Corridors can be used as a reliable guard to prevent fungal contamination in patient rooms. Only buildings equipped with HEPA filters achieved adequate air quality.
Rudramurthy *et al*. [33] 2016	Cross-sectional	Fungi	Emergency ward General medical ward	**(I)**: Common AC **(C)**: Natural ventilation	The average counts of *Aspergillus* spp. isolated from the non-AC area were significantly higher (*P* = 0.013) than for AC area. *A. flavus* and *A. fumigatus* were the most common species isolated	Except for *Aspergillus* spp., there was no significant difference in the average spore counts of other fungi in the AC and non-AC areas during different seasons.
Bozic *et al*. [34] 2019	Cross-sectional	Bacteria and fungi	Wards	**(I)**: Common AC **(C)**: Natural ventilation	The average levels of bacteria (587 CFU/m^3^) and fungi (308 CFU/m^3^) indicated that all hospital rooms were generally contaminated. Higher counts were found in areas without AC systems.	AC systems are effective in reducing microbiological contamination. Bacterial and fungal counts and microclimatic parameters with an AC system indicates regular maintenance.

Authors	Study	Microbes/Infection	Locations	Air-conditioning systems ((I):intervention; (C): control)	Key findings	Conclusions
Jung *et al*. [35] 2015	Cross-sectional	Bacteria	Nurse stations Pharmacy departments Clinics and waiting areas Lobbies Meeting rooms Wards	**(I)**: Common AC (Window type or single-split type) **(C1)**: Air Handling Unit (AHU) **(C2)**: Fan Cooling Unit (FCU) **(C3)**: AHU mix FCU	Carbon monoxide levels were higher in hospitals with AHU mix FCU compared to those with window or single-split type. Levels of PM2.5, PM10 and fungi were higher in hospitals with window or single-split type than with central air conditioning. Ratios of indoor to outdoor (I/O) concentrations for PM10, PM2.5 and fungi were mostly <1 in all settings; I/O ratios for fungi were > 1.0.	Central air conditioning (AHU/FCU) is more effective for the removal of aerosol pollutants than non-central air conditioning.
Hansen *et al*. .[36] 2008	Cross-sectional	Bacteria and ultra fine particles	Workplaces Wards	**(I)**: Common AC **(C)**: Natural ventilation	Concentrations of particles ≥0.5 mm (*P* = 0.04) and ultrafine particles (*P* = 0.001) were significantly lower in workplace rooms with additional ventilation on and air-conditioning systems than in rooms without. In rooms without ventilation and air-conditioning systems bacterial concentrations (*P *≤ 0.001) and particles ≥0.5 mm (*P* = 0.011) and ≥5 mm (*P *≤ 0.001) were significantly higher with window ventilation (*n* = 10) than not (*n* = 6).	Window ventilation leads to higher particle but not bacterial concentrations than HVAC systems. Concentrations of particles were significantly lower in air of rooms with additional ventilation and air-conditioning systems

Authors	Study	Microbes/Infection	Locations tested	Air-conditioning systems used ((I): intervention; (C): control)	Key findings	Conclusions
Hahn *et al*. [37] 2002	Retrospective/cohort	*Aspergillus*	Wards Workplaces	**(I)**: Common AC **(C)**: With HEPA filters	Air sampling showed that wards with HEPA filters had relatively low conidia counts (<4 CFU/m^3^), compared with those without such filters (>100 CFU/m^3^).	HEPA filters are effective at removing most of *Aspergillus* conidia from ambient air and are protective for highly immunocompromised patients with haematologic malignancies.
Ostojic *et al*. [38] 2017	Prospective/cohort	Invasive aspergillosis (IA)	Wards	**(I)**: Common AC **(C)**: With HEPA filters	No significant difference was found in the occurrence of IA between groups of patients treated in rooms with (*n* = 52), and without (*n* = 56), HEPA air conditioning. Likewise, for the median time to IA development (24.5 and 23.5 days, respectively).	HEPA air conditioning did not appear to impact on the occurrence, or median time to development of IA.
Takuma *et al*. [39] 2011	Cross-sectional	Fungi Invasive pulmonary mycoses (IPM)	Communal/Private wards	**(I)**: Common AC **(C)**: LAF system	Nosocomial IPM occurred more frequently during seasons in which the HVAC systems were not in use. Female patients in private rooms had lower rates of fever after sealing and disuse of air-conditioning systems (OR 0.0016; 95% CI 0.000–0.209).	Moulds colonising HVAC systems may have adverse effects on patients health, but definitive evidence is lacking.

Authors	Study	Microbes/Infection	Locations	Air-conditioning systems used ((I):intervention; (C): control)	Key findings	Conclusions
Nair *et al*. [40] 2017	Cross-sectional	Bacteria and fungi Hospital-acquired infections (HAI)	Wards	**(I)**: Common AC **(C)**: Natural ventilation	Highest prevalence of HAI was found in wards with central AC (22.72%), followed by split AC (11.43%). Odds of acquiring an HAI were 8.59 times more in patients exposed to central AC, and 4.20 times more to split AC. A significant association between AC and HAI (*P* < 0.05) was evident for all modalities of ventilation.	Exposure to central air-conditioning (OR 8.59) had higher odds of acquiring HAI (*P* < 0.05).
Silva *et al*. [41] 2010	Case control	Viruses	Emergency room	**(I)**: Common AC **(C)**: Natural ventilation	Respiratory viral infection occurred in 8.9% of patients admitted to the emergency within in the previous 5 days. Exposure to air conditioning was significantly associated with those with viral infection (*n* = 9, 40.9%) than without viral infection (*n* = 25, 14.9%) (*P* = 0.006).	Air conditioner use was the only factor associated with onset of respiratory viral infections.

Authors	Type of study	Outcome of interest	Hospital locations tested	Air-conditioning systems used ((I): intervention; (C): control)	Results concerned with air-conditioning systems	Conclusion
Jiamjarasrangsi *et al*. [42] 2009	Cross-sectional	Tubercle bacillus	Patient-care and ancillary areas	**(I)**: Common AC (Window or split type) **(C1):** Common AC (Central type) **(C2)**: Natural ventilation	Tracer gas measurements of air changes per hour for natural ventilation, central air conditioning and window or wall-mounted air conditioning areas were respectively 31.0 (51.4; *P* < 0.001), 12.6 (51.4); *P* < 0.05 and 2.7 (9.1); *P* < 0.001).	Ventilation rates in indoor work areas with air conditioning especially window or wall-mounted less efficient than natural ventilation. Inadequate ventilation is a major contributory factor to the spread of TB in nosocomial outbreaks.

Air-conditioning systems (AC) used in each area were categorised as ‘Common AC,’ ‘Natural ventilation,’ or ‘With HEPA filters’ if other details of ventilation were not given. Specific parameter description such as the type of air conditioner was recorded if given.

### Concentration of microorganisms in indoor air

Of the 21 studies included, 16 reported the concentrations of microorganisms in air samples from rooms (wards, corridors, laboratories) with different air-conditioning systems; five of the 16 also sampled outdoor hospital sites. Seven studies [22,26,27,29,33,34,36] reported results of microbial concentrations between naturally ventilated, and areas with common air-conditioning systems. In public areas of the hospitals, fungal loads in air-conditioned areas were considerably lower than those recorded in other indoor naturally ventilated environments [26,27,29,33,34]. Moreover, the average levels of bacteria were similar to those recorded for fungi [22,34,36], the latter being most probably derived from the outdoor environment of the hospital [27].

Air-conditioning systems were further classified into those with, or without HEPA filters in 10 studies. Compared to rooms without air-conditioning or with natural ventilation, indoor airborne fungal and bacterial concentrations were the lowest in rooms with HEPA filters, thus demonstrating their effectiveness for the reduction of bioaerosols [22,24,26,28,29,31,32,37]. Furthermore, the type of air conditioner used was considered crucial as central air conditioners proved to be more effective than non-centrally sited systems such as window, or single-split types [35,36]. Notably, one study identified that, compared with hybrid ventilation, the concentration of indoor bioaerosols was positively correlated with the type of ventilation system used (e.g., central air conditioners, *P* < 0.05) [23].

### Ratios of viable microorganisms detected

Four studies analysed associations between air-conditioning and the rates of viable microorganisms, mainly *Aspergillus*, detected on sampling [22,28,30,39]. The proportion of air samples positive for *Aspergillus* was consistently much higher in rooms in which the air-conditioning systems were not in use at the time of sampling [22,28,39]. However, the lowest mean recovery rate, and percentage of samples positive for *Aspergillus* were recorded in another study in areas with HEPA-filtered air-conditioning systems. In contrast, the samples collected from patient care areas without HEPA-filtered systems and the other reference samples were almost indistinguishable in terms of mean counts of *Aspergillus* spp. [30].

### Related infectious diseases

Five studies (two cross-sectional, two cohort and a case−control) reported outbreaks of related infectious diseases in air-conditioned hospitals. Exposure to central air-conditioning (OR 8.59) had a higher probability of causing hospital-acquired infections (*P* < 0.05) [40]. In addition, 8.9% of the patients admitted to the emergency room with onset of respiratory symptoms had viral infections, and exposure to air-conditioned air was the only linking factor [41]. Moreover, nosocomial invasive pulmonary mycoses occurred more frequently during seasons in which the HVAC systems were not in use than when they were used [39]. Only one study reported that the occurrence of invasive aspergillosis (IA) and median time to onset of infection was not significantly different between groups of patients treated in areas with, and without, HEPA-filtered air-conditioning [38]. However, another study indicated that HEPA filters were protective for highly immunocompromised patients with haematologic malignancies and were effective for removing most *Aspergillus* conidia from the ambient air [37].

### Microbe species

Only five studies reported on specific identification of microbial species, mainly fungi, in air-conditioned hospitals. A cross-sectional study in a South Korean hospital [29] assessed the degree of fungal contamination in hospital air environments over the course of a year, and found that *Aspergillus* spp. were the most prevalent both inside (47.0%) and outside (62.0%) the hospital. Within the hospital, *Penicillium* spp. were the second most predominant fungi, accounting for 37.9% (*n* = 25) of the identified species and 8.9% (*n* = 14) of those found outside (*P* < 0.001). Overall, the third most common moulds were of the *Alternaria* genus [29]. Similar results were reported in another cross-sectional study in 10 hospitals by Perdelli *et al*. [26], which found that the mean concentrations of *Aspergillus*, *Penicillium*, *Cladosporium* and *Rhizopus*, which were implicated in patient's infections, were significantly higher in the kitchens than in other tested areas with HEPA filters in the air-conditioning systems.

In another study, samples of airborne fungi at a tertiary university hospital were collected monthly over 10 years, and all *Aspergillus* isolates were further categorised into different species, namely, *A. fumigatus, A. niger* and *A. flavus*; the latter two species were the most prevalent [30]. Likewise, in another study, *A. flavus* and *A. fumigatus* were the most common species isolated in rooms with or without air conditioners. The average number of *Aspergillus* spp. isolated from the non-air-conditioned rooms was significantly higher than from air-conditioned areas (*P* = 0.013) [33].

### Indirect factors

Evidence of indirect factors influencing the effectiveness of air-conditioning was provided through a cross-sectional survey of 323 patient care, and ancillary areas, in hospitals of Thailand. This found that indoor ventilation rates (air changes per hour) of areas with central air-conditioning (median, 12.6) were consistently lower than those of work areas with natural ventilation (median, 31.0) (*P* < 0.001). Furthermore, the ventilation rates of areas with window or wall-mounted air conditioners (median, 2.7) were significantly less than in centrally air-conditioned areas (*P* < 0.001) [42]. Patients in rooms with low ventilation rates might have a higher risk of getting infected by the spread of *Mycobacterium tuberculosis* [43].

## Discussion

Scoping reviews aim to show the primary resource and types of available evidence to provide key concepts for clinical practice, policy formulation and research, especially in an area which has not been reviewed systematically [21].

In this study, we reviewed the relevant literature to assess how air-conditioning systems affect the incidence and impact of pathogenic microorganisms in the public indoor areas of hospitals. Air-conditioning systems play a more important role than heating or cooling the air in hospitals and other healthcare environments. A hospital is a public setting visited by various kinds of patients from different places. Thus, the issue of microbial contamination related to the use of air-conditioning systems cannot be underestimated, especially given the ongoing COVID-19 pandemic.

The review identified that, in public areas of hospitals, bacterial and fungal bioaerosol concentrations were generally higher in naturally ventilated rooms compared with the degerming effect of central air-conditioned systems which are proven to be effective in removing airborne microbes, although fungal spore levels may remain high in air-conditioned rooms [33]. The latter reinforces the need for periodical maintenance and disinfection of air-conditioning systems to prevent environmental colonisation and dissemination of fungi [33,34]. Evidence suggests that patients exposed to air-conditioning systems had higher risks of acquiring a viral, or hospital-associated bacterial or fungal infection, the latter potentially causing invasive pulmonary mycoses. Moreover, when air-conditioning systems were in use, doors and windows were often closed to maintain a suitable temperature, which resulted in reduced ventilation rates [42]. Likewise, poor design and operation of air-conditioning systems can contribute to inadequate ventilation [44,45] and these factors may account for the increase in infection risks when exposed to air-conditioning systems in hospitals. Compared to window or split types of air-conditioning systems, often used in single-patient rooms, recycled central air-conditioning systems were more often installed in multiple-patients' room in a study conducted in a certain hospital in India [40]. Contact between patients and increased movement of personnel may also contribute to higher risk of acquiring hospital infections when exposed to central air-conditioning systems.

To the best of our knowledge, this is the first review in which the influence of high-efficiency filters in air-conditioning systems on the spread of microorganisms has been evaluated. Our key finding is that filters appear to be an indispensable part of air-conditioning systems. Ten of the studies addressed the benefits of HEPA filters in these systems and clearly showed that the concentration of airborne microorganisms in areas with HEPA filters was lower than the concentration in areas without them. However, the included studies did not focus on the non-HEPA filters that are commonly installed inside air conditioners, and few provided details of the operating system, such as pressurisation, humidity, temperature etc. Two studies reported on the efficiency of their non-HEPA filters used in the areas tested [30,37]. Indeed, only one gave details of the mean temperatures and relative humidity of the natural ventilated areas and in the air-conditioned areas [29]. These factors may be the source of the heterogeneity of data noted in studies that simply classified areas based on the presence of an air conditioner or did not specify the type of air conditioners.

In a workshop summary of the Institute of Medicine (US) Forum on Microbial Threats, HEPA was defined as a pleated mechanical air filter composed of mats of randomly arranged glass fibres that collects and traps particles greater than 0.1 μm by diffusing, intercepting and impacting the passage of particles [46]. A study conducted in two Wuhan hospitals showed that SARS-CoV-2 aerosols were mainly found in the submicrometer areas (aerosol size distributions between 0.25 and 1.0 μm) and supermicrometer areas (aerosol size distributions > 2.5 μm) [47]. Air filtration through HEPA can intercept most pathogens, including fungi, bacteria and encapsulated viruses, with an efficiency >99.97% [46]. Although direct studies for SARS-CoV-2 have not as yet been performed, the current study on HEPA filter functionality, and prior CDC guidelines for SARS-CoV-1 together suggest a theoretical efficacy for HEPA filters in eliminating airborne SARS-CoV-2 [48].

HEPA filters in air-conditioning systems are widely acknowledged to be highly effective for the removal of microorganisms from the air and protective for high-risk patients. However, owing to their high costs of installation and maintenance, it may prove difficult for healthcare facilities to fit air-conditioning systems with HEPA filters in isolated areas, let alone in public areas. Even in the United Kingdom, only a quarter of 203 hospitals surveyed had isolation facilities available in their emergency departments [49]. This situation could only be worse in low-income and developing countries. Nevertheless, a cost-effectiveness incremental analysis showed that for prevention of invasive aspergillosis, rooms with HEPA-filtered systems were better cost-saving interventions than antifungal (posaconazole) prophylaxis and environmental protection measures ($2665 *vs.* $ 42 531 *vs.* $4073, respectively) [50], and thus the economic benefits of such filters can exceed the costs of installation and maintenance.

For areas where HEPA filters are currently not available, possible substitutes to improve air hygiene are: lamps with germicidal ultraviolet irradiation, increasing room ventilation rates, and less widely applied, generation of hydrogen peroxide mists stabilised with silver ions [51–56]. Microbial contamination of room air and risks of transmission can be reduced to a minimum by regular implementation of disinfection measures. For hospitals in poor areas or with inadequate external air quality, mobile air-decontamination units and portable HEPA filtration units are alternative options and are easy to maintain [57,58].

This scoping review has some limitations. First, all the included studies reported different descriptions of the air-conditioning systems used, which may be responsible for differences in their conclusions. Second, although several studies provided seemingly detailed descriptions of the air sampling methods used, variables in the experimental set-up were not described. Details of the sampling time, and the position and height of the sampler when samples were taken, were generally imprecise or not reported. Third, locations of the hospitals, humidity, temperature and season have recognised impacts on microbial contamination of indoor air [24,29,59]; these factors were considered in relatively few of the studies. Lastly, as standard deviations of microbe concentrations were reported inconsistently, the data presented may therefore be an underestimation of reality since the sampled areas were not randomly selected. Further, specific microorganisms in various settings were assessed based on selective sampling and reliance on existing techniques; thus, other microbes in the air and on surfaces might have been overlooked. Nevertheless, we consider that these limitations do not affect the validity and conclusions of the study.

In conclusion, this study focused on ventilation of hospital public areas, which are more likely to be overlooked relative to operating room and ICUs, and reviewed evidence regarding the risk of air-conditioning systems and hospital-acquired infections. The cleaning and maintenance of such systems should be done regularly according to existing standards as patients residing in contaminated air in rooms have a higher risk of exposure to pathogenic microorganisms. The universal installation of HEPA filters can effectively mitigate against microbial contamination and constitute a protective measure for patients. These findings may help improve management of air-conditioning systems during a pandemic. Future studies should attempt to assess multiple air-conditioning parameters during operational hours with quantitative and qualitative measurements of temperature, relative humidity and ventilation rates.

### Strengths and limitations of this study

- Systematic methods were used to provide a comprehensive review of effects of air-conditioning systems and HEPA filters on the transmission of pathogenic microorganisms and related diseases.
- This study focused on hospital public areas, which are more likely to be overlooked relative to areas such as the operating room and ICU.
- Only articles published in English were included in this study.

## Data Availability

Data available on request due to restrictions.

## Supplementary material

For supplementary material accompanying this paper visit http://dx.doi.org/10.1017/S0950268821001990. click here to view supplementary material
