# Supplementary material for: Effects of air-conditioning systems in the public areas of hospitals: A scoping review
Source: Epidemiol Infect. 2021 Aug 27;149:e201. doi: 10.1017/S0950268821001990 (PMC8438419; doi:10.1017/S0950268821001990)
Supplement: Supplementary file 1 [file hygsup.zip › S0950268821001990sup001.docx]

**supplementary material:Table 1. Search strategy**

| # | Search Strategy |
| --- | --- |
| 1 | 'built environment'/exp/mj |
| 2 | 'air conditioning'/exp/mj |
| 3 | 'air condition':ab.ti OR 'air conditioner':ab.ti OR 'air conditioning':ab.ti OR 'ventilation system':ab.ti OR |
|  | 'air handing system':ab.ti OR 'building system':ab.ti OR 'heating system':ab.ti OR 'cooling system':ab.ti OR |
|  | 'humidification system':ab.ti OR 'air revitalization':ab.ti OR 'cooling center':ab.ti OR 'air delivery systems':ab.ti OR |
|  | 'built environment':ab.ti OR 'climate control':ab.ti OR 'hvac system':ab.ti |
| 4 | #1 OR #2 OR#3 |
| 5 | 'hospital'/exp/mj |
| 6 | hospital:ab,ti |
| 7 | #5 OR#6 |
| 8 | #4 AND#7 |
| 9 | 'microorganism'/exp/mj |
| 10 | 'virus'/exp/mj |
| 11 | 'bacterium'/exp/mj |
| 12 | 'fungus'/exp/mj |
| 13 | 'mycoplasma'/exp/mj |
| 14 | 'chlamydial'/exp/mj |
| 15 | 'coronavirus disease 2019'/exp/mj |
| 16 | 'sars coronavirus'/exp/mj |
| 17 | 'middle east respiratory syndrome coronavirus'/exp/mj |
| 18 | 'coronaviridae'/exp/mj |
| 19 | 'avian influenza'/exp/mj |
| 20 | 'tuberculosis'/exp/mj |
| 21 | 'measles'/exp/mj |
| 22 | 'legionella'/exp/mj |
| 23 | 'aspergillus'/exp/mj |
| 24 | 'aspergillus'/exp/mj |
| 25 | 'fever'/exp/mj |
| 26 | 'acute respiratory tract disease'/exp/mj |
| 27 | 'aspergillus'/exp/mj |
| 28 | 'bacterium'/exp/mj |
| 29 | microorganism:ab,ti OR virus:ab.ti OR bacteria:ab.ti OR fungi:ab.ti OR fungus:ab.ti OR. mvcoplasma:ab.ti |
|  | OR chlamydia:ab.ti OR germ:ab.ti OR 'covid 19':ab.ti OR sars:ab.ti OR mers:ab.ti OR coronavius:ab.ti OR 'avian |
|  | influenza':ab.ti OR tuberculosis:ab.ti OR measles:ab.ti OR legionella:ab.ti OR Influenza:ab.ti OR fever:ab.ti OR |
|  | 'respiratory disease':ab.ti OR aspergilla:ab.ti OR aspergillus:ab.ti OR bacterium:ab.ti |
| 30 | #9 OR #10 OR #11 OR #12 OR #13 OR #14 OR #15 OR #16OR #17 OR #18 OR #19 OR #20 OR #21 OR #22 OR #23 OR |
|  | #24OR #25 OR #26 OR #27 OR #28 OR#29#31#8 AND#30 |

Symbols are field labels according to Embase: ab, Abstract; ti, Article title; exp, Emtree term-exploded; mj, Limit to terms indexed in articles as ‘major focus’.
