# Supplementary material for: Effects of air-conditioning systems in the public areas of hospitals: A scoping review
Source: Epidemiol Infect. 2021 Aug 27;149:e201. doi: 10.1017/S0950268821001990 (PMC8438419; doi:10.1017/S0950268821001990)
Supplement: Supplementary file 1 [file hygsup.zip › S0950268821001990sup002.docx]

**supplementary material:TABLE 2 Outcomes of the included studies**

|  | Concentration of indoor air microorganisms | Detecting ratio | Microbe species | Related infectious diseases |
| --- | --- | --- | --- | --- |
| Perdelli, 2006(A) [22] | ✔ | ✔ |  |  |
| Sornboot, 2019 [23] | ✔ |  |  |  |
| Çakir, 2013 [24] | ✔ |  |  |  |
| Crimi, 2009 [25] | ✔ |  |  |  |
| Perdelli,006 (B)[26] | ✔ |  | ✔ |  |
| Lidwell, .1975 [27] | ✔ |  |  |  |
| Crimi,2006 [28] | ✔ | ✔ |  |  |
| Cho, 2018 [29] | ✔ |  | ✔ |  |
| Falvey, 2007 [30] | ✔ | ✔ | ✔ |  |
| Kruger, 2003 [31] | ✔ |  |  |  |
| Bellanger, 2017 [32] | ✔ |  |  |  |
| Rudramurthy, 2016 [33] | ✔ |  | ✔ |  |
| Bozic,2019 [34] | ✔ |  |  |  |
| Jung, 2015 [35] | ✔ |  |  |  |
| Hansen, 2008 [36] | ✔ |  |  |  |
| Hahn, 2002 [37] | ✔ |  |  | ✔ |
| Ostojic, 2017 [38] |  |  |  | ✔ |
| Takuma, 2011 [39] |  | ✔ |  | ✔ |
| Nair, 2017 [40] |  |  |  | ✔ |
| Silva, 2010 [41] |  |  |  | ✔ |
| Jiamjarasrangsi, 2009 [42] |  |  |  | ✔ |
